# Relative heritage language and majority language use before school start explains variance in 2^nd^ grade majority language but not reading skills

**DOI:** 10.3389/fpsyg.2023.1134830

**Published:** 2023-04-17

**Authors:** Anders Højen, Dorthe Bleses

**Affiliations:** ^1^Department of Linguistics, School of Communication and Culture, Aarhus University, Aarhus, Denmark; ^2^TrygFonden’s Center for Child Research, Department of Economics and Business Economics, Aarhus University, Aarhus, Denmark; ^3^Centre for Integrated Register-Based Research, Department of Economics and Business Economics, Aarhus University, Aarhus, Denmark

**Keywords:** bilingualism, heritage language use, minority language use, home literacy environments, socioeconomy, reading outcomes, literacy, reading development

## Abstract

The present study examined whether parents’ and bilingual children’s own relative use of the heritage language vs. the majority language in the homes of bilingual children in Denmark before school start explains variance in 2^nd^ grade majority language skills and reading skills. The study included two groups of children: the Mixed bilinguals group (defined by having a native Danish and a nonnative parent, *N* = 376) and the Heritage bilinguals group (defined by having parents who were both speakers of a Heritage language, *N* = 276). Four-stage hierarchical regression analyses showed that, after accounting for type of bilingualism, socioeconomic status (SES) and home literacy environment quality, relative use of the heritage vs. the majority language explained variance in 2^nd^ grade Danish language comprehension scores, but did not explain variance in two reading scores, namely decoding and reading comprehension. In addition, a home literacy factor denoting book exposure (number of books, frequency of reading, library visits, and age of beginning shared book reading) was a significant predictor of both 2^nd^ grade language and reading outcomes, whereas SES became a nonsignificant predictor when adding home literacy and language use predictors. We interpret the results to mean that parents’ and the child’s own relative use of the heritage language vs. the majority language before school start does not influence bilingual children’s early reading skills, whereas a supportive early home literacy environment is a positive predictor of reading skills independently of SES and parental majority language use and skill.

## 1. Introduction

Having good language and reading skills in the early years of school is an important foundation for academic achievement (e.g., Rabiner et al., 2016). In turn, those skills are predicted by early language and preliteracy skills children already before school start (e.g., Snow et al., 1998; Whitehurst and Lonigan, 1998; Bleses et al., 2016; Dale et al., 2023). However, many children are faced with the task of learning not only the language of schooling, the majority language, but also a heritage language, often because one or both parents immigrated to another country. Because of the importance of skills in the language of schooling, parents of young bilingual or bilingual-to-be children may wonder whether their children are best supported by parents minimizing use of the heritage language in the home and prioritize the majority language. Studies often show that bilingual or immigrant children have lower academic achievement than other children, but this population also typically differs on other potentially important factors that have been shown to be correlated with language development and academic achievement, notably family socioeconomic status (SES; White, 1982) and the home literacy environment (Zauche et al., 2016; Højen et al., 2021). Therefore, the purpose of the present study was to determine whether variance in proportion of heritage language vs. majority language (Danish) use in the homes of 3–6-year-old bilingual children in Denmark explains variance in later Danish language and reading skills in 2^nd^ grade.

### 1.1. Early predictors of language and reading kills in school

Language and reading skills in the first years of school are predicted by early oral language skills, such as vocabulary size and language comprehension, as well as preliteracy skills, such as phonological awareness and familiarity with print and letters (Snow et al., 1998; Whitehurst and Lonigan, 1998; McBride-Chang and Kail, 2002; Gonzalez et al., 2011; Bleses et al., 2016). Relationships of early skills in the language of schooling to later reading skills are found also in bilingual children (August and Shanahan, 2006; Demie and Strand, 2006; Kieffer, 2008; Halle et al., 2012). For example, Halle et al. (2012), in a large sample of almost 20,000 U.S. school children, including 2,700 language-minority children, examined educational outcomes as a function of when language minority students achieved English proficiency. They found that, after accounting for a range of control variables, language-minority children who were proficient in English at school start were able to keep pace with native English speakers in terms of educational outcomes. On the other hand, those who achieved English proficiency relatively late in school continued to have educational gaps with native speakers in reading (and even more so in math), and those who were not English proficient at school start but reached English proficiency in 1^st^ grade showed intermediate outcomes.

The positive association between early majority language proficiency and educational outcomes might lead to the conclusion that parents of bilingual children ought to focus on majority language development and prioritize speaking the majority language over the heritage language in the home. However, the relationship of parents’ use of heritage vs. majority language to later outcomes varies with outcome. For example, Winsler et al. (2014), in a study of children in kindergarten who experienced different degrees of heritage language use in the home, found that parents’ use of the heritage language in the home was positively related to early cognitive/language development (Bayley measures) and math skills at school start. On the other hand, there were no reliable relationships of parents’ degree of heritage language use to early literacy skills at kindergarten entry.

Thus, the results of Winsler et al. and Halle et al. indicate at the same time at that early majority language skills are important for educational outcomes and that use of the heritage language with the child in early childhood does not hinder favorable educational outcomes later in school. These results suggest a complex relationship of parents’ early language use, children’s language skill, and other factors to later reading skills. A possible reconciliation of the seemingly conflicting results is that parents’ *absolute* use (i.e., minutes of daily use) of the majority language and the heritage language with bilingual children is more important than *relative* use (i.e., the percentage use of each language). In other words, children need rich interactions in whatever language parents can best provide those interactions in order to develop good language skills early in life and later reading skills, as suggested by Giguere and Hoff (2023). In support of this proposition, Mesa and Yeomans-Maldonado (2019) found that early oral skills in the heritage language had direct predictive relationships to reading skills in the majority language. If so, this would suggest that the timing of the onset of majority language/second language acquisition and parents’ degree of use of the majority language are not of decisive importance for later majority language reading skills as long as the child gets stimulating language exposure. However, parents’ degree of majority language use and timing of onset of majority language acquisition are two different dimensions.

### 1.2. Simultaneous and sequential bilingualism, language use, and language skills

Some bilingual children begin to acquire two languages at home from the beginning of life, often because one of the parents speaks a heritage language and the other parent speaks the societal majority language as a native language. Other bilingual or bilingual-to-be children grow up acquiring a heritage language at home and a second language, the majority language, predominantly outside of the home, at the latest when entering school. This is typically when both parents are native speakers of a language other than the majority language. Those two types of bilingualism are often referred to in the literature as simultaneous and sequential bilingualism, respectively. However, in a Danish context, where the present study was conducted, it is most common for children to enter childcare at about 12–15 months of age, which means that even bilingual children with two nonnative parents begin to be exposed to the majority language well within the age normally denoting simultaneous bilingualism (often tentatively set at <3 years of age). Therefore, to avoid confusion, we will later refer to bilingual children *in Denmark* with mixed Danish and heritage language parents as “Mixed bilinguals” and to bilingual children with two heritage language parents “Heritage bilinguals.”

Both heritage and majority language acquisition develop predictably as a function of quantity of exposure to each language in the home. This has been found for several linguistic domains, for example receptive vocabulary, expressive vocabulary, processing speed, and morphology (Umbel and Oller, 1994; Hoff, 2018; Thordardottir, 2019). Simultaneous bilinguals have *earlier* exposure to the majority language in their homes than sequential bilinguals do, but given that simultaneous bilinguals receive majority language input in the home, they are also likely to receive *more* majority language input than sequential bilinguals in the preschool age. That is, simultaneous bilinguals will have a double advantage when entering school by having received majority language exposure in higher proportions and for a longer time compared to sequential bilinguals. But which is more important further downstream in majority language development, high amounts or early onset of majority language input?

A study pitting amount of majority language exposure in the home against timing of exposure (age of first exposure) found that “amount trumps timing” in 1^st^ and 3^rd^ grade native English learners of French with respect to receptive and expressive vocabulary and morphology skills (Thordardottir, 2019). This was because the simultaneous vs. sequential bilingual group differences became nonsignificant when controlling for amount of exposure. However, when controlling for timing of exposure (but not amount of exposure), group differences remained significant for receptive vocabulary in 1^st^ graders and for expressive vocabulary.

This finding of the importance of *quantity* of majority language exposure in the home environment seems to be at odds with the findings by Winsler et al. (2014), which suggested that parents’ relative use of the heritage and majority language did not significantly influence later reading. However, note that Thordardottir (2019) measured majority *language* outcomes while by Winsler et al. (2014) measured early reading outcomes. It is conceivable that early reading is less impacted by degree of use of the majority language in the home because an important component of early reading is decoding skills. Decoding skills draw on phonological awareness, a skill that is not negatively influenced—but possibly positively influenced—by bilingualism (Hammer et al., 2014). Moreover, reading-related skills in general transfer better between languages than do oral language skills (Cummins, 1991; Adesope et al., 2010; Hammer et al., 2014).

At least one more factor complicates interpretation of relationship of parental language use (heritage vs. majority language) to child language development, namely parental language skill. If parents are a nonnative speakers, their skill level in the majority language will vary considerably, and their speech in child interactions may contain fewer of those lexical and grammatical properties that have been shown to positively predict child language development (Hoff et al., 2020). Therefore, nonnative parents with relatively low majority language skills may do their child a disservice by speaking the majority language rather than provide a richer heritage language model to help their child’s heritage language development. Additionally, the child’s own language use matters. A common pattern in bilingual families is that the child replies in the majority language even if the parent addresses the child in the heritage language. Children’s degree of use of the majority language has been shown to predict *expressive* majority language growth over and above effects of majority language exposure, whereas children’s majority language use did not predict language *comprehension*; only children’s language exposure predicted comprehension (for a review, see Hoff et al., 2022).

The finding of the importance of parents’ relative language use and skills for oral language development in bilingual children is in line with the convergence of bilingual research on usage-based accounts of bilingual language development indicating that language acquisition is a general cognitive process greatly influenced by language use and experience (Ellis, 2002; Hernandez et al., 2005; MacWhinney, 2005; Højen, 2019). These findings are also related to the important observation that monolinguals should be expected to function as “two monolinguals in one person” (Grosjean, 1989). However, factors other than relative use and skill have been shown to influence language development in bilingual children.

### 1.3. Relationship of socioeconomic status to language and reading outcomes

A ubiquitous factor in child development is family socioeconomic status (SES). SES is typically indexed by parental education level and income, and these factors have also been shown to be related to language and reading development (Ginsborg, 2006; Rowe et al., 2016). This is particularly worrying in the context of bilingualism because immigrant populations often have lower SES than non-immigrant populations, although this varies greatly across different immigrant populations and host countries (Dustmann et al., 2012). Moreover, in the context of Denmark, where the present study was carried out, we recently found evidence that the association between SES and young children’s language/preliteracy outcomes was significantly *stronger* in some immigrant populations than in non-immigrants (Højen et al., 2019).

It is, of course, not parental income or education *per se* that influences children’s language and reading outcomes. Part of the mechanism that transfers SES effects from parents to children has, as mentioned, been identified as parental language skill, and thereby the language models that they can provide for their children (Sullivan et al., 2021). In addition, the overall home literacy environment has been shown to mediate SES relationships to vocabulary development (Singh et al., 2022), and home literacy environments may be a stronger predictor of children’s language and reading outcomes than traditional SES variables, income and education (Højen et al., 2021).

### 1.4. Relationship of home literacy environments to language and Reading outcomes

The home literacy environment traditionally refers to tangible literacy related resources in the home such as books or letters to play with, as well as language-and literacy-oriented parent practices with the child, such as shared book reading, nursery rhymes, and singing (Sénéchal et al., 1998; Foy and Mann, 2003). In addition, after the last decade’s surge in use of mobile screen media, the nature of the digital home literacy and its effect on child development have gained interest in recent years (Segers and Kleemans, 2020; Turco et al., 2023). When many of children’s experiences with child literature and exposure to literacy come from mobile screen devices, it is clear that this is an important new aspect of the home literacy environment. This is particularly interesting in the context of bilingual children; children’s books and literacy materials may not be easily available in the heritage language of bilingual families but may become accessible digitally. However, although Segers and Kleemans (2020) found that the digital and analogue home literacy environments constituted different factors, the digital factor was not related to child language outcomes. A similar finding was reported by Turco et al. (2023), who found a simple *negative* association between child use of digital media and their language and reading outcomes; the association, however, was driven by demographic characteristics of the family. Thus, since the literature on digital home literacy environments is only in its infancy, much more research is needed to document associations—positive or negative—with different aspects of the digital home literacy environment to child outcomes.

On the other hand, there is a well-documented association between the analogue home literacy environment (hereafter, just “the home literacy environment”) and children’s language and early literacy/reading development. This association has been found in both monolingual children (Sénéchal et al., 1998; Foy and Mann, 2003) and bilingual children (Sénéchal and LeFevre, 2014; Højen et al., 2021) and mixed monolingual/bilingual samples (Segers and Kleemans, 2020). In addition, Højen et al. (2021) found that the association between the home literacy environment and language outcomes was *stronger* in bilingual 4–6-year-olds compared to their monolingual peers. This result points to the importance of a highly supportive environment for bilingual children, whose language acquisition task is doubled. However, at the same time, the study found that home literacy environments of sequential bilinguals were substantially poorer (about 0.30 to 1.25 standard deviation depending on the measure) than those of Danish monolingual children, whereas the quality of the home literacy environments of simultaneous bilinguals was very similar to those of Danish monolinguals. This difference could be related to a lower SES on average in parents of sequential bilinguals (both parents being immigrants) and/or to unavailability of books and other literacy materials in the heritage languages.

### 1.5. The present study

In summary, previous research shows that early language skills are related to later language and reading skills in school in bilingual children as well as monolingual children. Early majority language skills, as well as heritage language skills, in bilingual children are predictably related to degree of parents’ use of the majority language vs. the heritage language in the home, their language skills and the child’s own degree of use of the majority language (although only expressive skills). However, while longer-term reading outcomes in the majority language are related to early majority language skills (Halle et al., 2012), they may not be related to degree of parents’ early language use of the majority language in the home (Winsler et al., 2014). This draws a pattern of complex predictors of reading skills in bilingual children and raises the possibility that parents speaking a heritage language can prioritize speaking the heritage language with the child in the home before school start without detrimental effects for the child’s later reading outcomes. However, other factors such as SES and the home literacy environments are also related to language and reading outcomes as noted earlier. And in a Danish context, heritage bilingual families (often having refugee background) have lower average SES and poorer home literacy environments than mixed bilingual families (Højen et al., 2021), which means that those factors should be controlled when examining the relationship of the child’s early language experiences in the home to later majority language and reading outcomes.

Therefore, the present study asks whether bilingual children’s language experiences (parents’ and child’s own majority language vs. the heritage language use as well as parent’s majority language skills) in the home before school start explain variance in children’s 2^nd^ grade majority language and reading scores after accounting for type of bilingualism (mixed vs. heritage bilinguals), family SES, and home literacy environments. Note that children’s own early language skills are not considered here, as we focus on the early language environment. Our specific research questions are:

1. Does degree of relative use of the heritage language and majority language in the home of preschool-age bilingual children explain a significant amount of variance in their 2^nd^ grade majority language and reading skills after controlling for type of bilingualism (mixed vs. heritage), family SES, and home literacy environment quality?
2. Are relationships of parental heritage language use in the home to bilingual children’s 2^nd^ grade majority language and reading skills moderated by type of bilingualism (mixed vs. heritage)?
3. Are relationships of parental heritage language use to bilingual children’s 2^nd^ grade majority language and reading skills moderated by parental majority language skill?

## 2. Materials and methods

### 2.1. Participants

The 652 bilingual children of the present study were 2^nd^ graders from 213 different Danish schools who completed a nationwide mandatory test battery of Danish language and reading skills in the years 2016–2018. Three to 5 years prior to the 2^nd^ grade test, when the children were in childcare, they were all enrolled in either of two parallel randomized control (RCT) studies in language and preliteracy intervention. Both were brief low-cost language and literacy interventions (20 weeks) nested in the usual childcare program with bi-weekly 30-min lessons (Bleses et al., 2018a,b). The original sample for the RCTs consisted of both monolingual and bilingual children. The present sample is a subsample of those children, namely children who (1) had one or two nonnative parents (2) had questionnaire information regarding home literacy environments and minority language use filled in by their parents at pretest of the original RCTs, and (3) had taken the language and reading test in primary school’s 2^nd^ grade. In each RCT, the children were either in a control group or in one of three intervention arms.

This sample consists of 376 children with a native Danish parent and a nonnative parent and 276 children with two nonnative parents. The questionnaire items pertaining to the native languages of the mother and father were used to classify children as either “heritage” bilinguals (both parents were native speakers of a heritage language) or “mixed” bilinguals (one native Danish speaking and one nonnative parent). In the abovementioned RCTs, the children had been either in a control group (Heritage bilinguals *N* = 54, Mixed bilinguals *N* = 100) or in one of three intervention arms (Heritage bilinguals *N* = 222, Mixed bilinguals *N* = 276). For Heritage bilinguals, the most frequent heritage languages were Arabic, Turkish, Yugoslavian (Serbian, Croatian, Bosnian), Kurdish, Somali, Urdu, and German. For Mixed bilinguals, the most frequent heritage languages of the nonnative parents were English, Polish, Russian, Thai, Kurdish, Arabic and German; the mother was the nonnative speaker in 60% of those children, and the father was the nonnative in the remaining 40%.

About 30% of the original, representative sample did not answer and submit the questionnaire, and non-responders had lower SES. The present subsample of bilingual questionnaire responders is thus not representative (higher SES), but because questionnaire information was used to classify participants as bilinguals, it cannot be determined exactly how bilinguals in our subsample differ from bilinguals not included (because of missing questionnaire information). However, given that responding to the multi-item questionnaire required a certain degree of literacy and Danish-language skills, parents lacking in those skills were necessarily underrepresented. Mean characteristics of the two participant groups are shown in Table 1.

**Table 1:** Basic mean characteristics of each bilingual group; *p*-value and *η*^2^ effect size for group differences (ANOVA).

	Heritage bilinguals N = 276 (56% boys)		Mixed bilinguals *N* = 376 (53% boys)		Group difference	
	Mean	SD	Mean	SD	*p*	*η* ^2^
Maternal education, years	12.7	3.5	15.2	2.7	<0.001	0.14
Household income, 100 K DKK	3.2	3.1	5.4	3.5	<0.001	0.10

As shown in Table 1, parents of Mixed bilinguals had higher SES than parents of Heritage bilinguals. In addition, although we do not have data documentation, it is very likely that a comparatively higher proportion of parents of Heritage bilinguals (two nonnative parents) had refugee background, whereas a comparatively higher proportion of parents of Mixed bilinguals (one native Danish and one nonnative parent) had work-or partner-related immigration backgrounds. Those differences together with the differences in the most frequent heritage language backgrounds, mean that the two groups of bilingual children differed not solely in whether or not they had the opportunity to learn Danish from a native parent in the home, which should be considered in the analyses following below.

### 2.2. Measures

#### 2.2.1. Maternal education and income

Two SES control variables were used: (1) maternal education measured in years of formal schooling in Denmark, and (2) gross house-hold income before any taxes, tax deductions, or welfare benefits. We obtained both measures from Statistics Denmark. Unfortunately, Statistics Denmark has reliable information only about degrees obtained in the Danish educational system, not about degrees obtained in the home countries. However, parental education obtained in the heritage language has previously been found to be predictive of children’s heritage language skills, whereas parental information obtained in the majority language of a host country has been found to be predictive of children’s second-language/majority-language skills (Hoff et al., 2018), which are the skills examined in the present study. In addition, a previous study, which included the present sample, found maternal education to be very predictive of bilingual children’s second language at age 4–6 (Højen et al., 2021). But total years of formal schooling is necessarily underestimated in parents who immigrated after having begun school.

#### 2.2.2. National tests in language and reading in 2^nd^ grade

The three outcome variables were 2^nd^ grade scores in an oral language test (Danish language comprehension) and two reading tests (word decoding and reading comprehension). The scores were obtained from a Danish national test battery for all 2^nd^ graders (Beuchert and Nandrup, 2017), and we were granted access to the scores via the national registry, Statistics Denmark.

The language comprehension test tests the understanding of Danish words, sentences, and proverbs in a multiple-choice format. The test is presented in written format, which means that there is a reading component in the skills required to complete the test. The decoding test tests the ability to identify possible Danish words by segmenting word strings, and the reading comprehension test tests the ability to read and understand a text by subsequently checking correct answers regarding the content of the text in a multiple-choice format. Students are assigned percentile scores, but presently we used standardized theta scores which are better suited for statistical analyses. Standardization was done on the whole population of 2^nd^ graders who took the test, which means that a score of zero corresponds to the national mean.

#### 2.2.3. Home literacy environments

Home literacy environments during the preschool years was measured via parental report prior to entering the above-mentioned language and preliteracy intervention studies. The questionnaire contained multiple items each rated on Likert scales, but presently we use the two home literacy environment factors identified in principal component analysis and used as predictors in previous research on the overall sample (i.e., including monolingual children; Højen et al., 2021, 2022). We use the standardized factor values obtained for the overall sample including also monolingual children. The items constituting the factor *book exposure* pertained to number of adults’ books in the home, number of children’s books, frequency of library and bookshop visits, frequency of shared book reading in the past week, and the child’s age when beginning shared book reading. The items constituting the factor *literacy activities* pertained to frequency of talking about letters, frequency of talking about numbers, frequency of singing with the child, and frequency of nursery rhymes and word plays.

#### 2.2.4. Heritage language and majority language use

The questionnaire filled in prior to the preschool RCT asked parents to rate both mother’s and father’s use of Danish vs. the heritage language in the home (5-point scale from *Mother language only (no Danish)* to *Danish only*), mother’s and father’s Danish language skills (5-point scale from *no skill at all* to *fluent*), and the child’s use of Danish in the home, in childcare, and when with friends (all three rated by parents on five-point scales from *no Danish* to *Danish only*). That is, the questionnaire examined *relative* use of the heritage language and Danish, but not *absolute use*.

### 2.3. Analytic strategy

Descriptive data analysis of all variables is first provided including zero-order correlations between predictors and outcome variables. Predictors that were significantly related to outcomes in the correlation analysis were retained as predictors of the three outcome variables in subsequent hierarchical regression models. The predictors were entered in blocks to examine the extent to which a block of language use variables and parent majority language skill variables explain variance in the three outcome variables (language comprehension, decoding, and reading comprehension) after accounting for type of bilingualism (Heritage vs. Mixed), SES (maternal education and household income), and home literacy environment variables. All analyses were carried out in STATA 15. STATAs *nestreg* function was used for the hierarchical regressions; standard errors were adjusted for clustering in schools.

## 3. Results

### 3.1. Descriptive statistics and preliminary analysis

Before examining the main questions of predictions of language and reading skills, this section gives basic descriptive statistics of the variables involved. Table 2 shows mean characteristics of the two bilingual groups. Because the 2^nd^ grade language and reading outcomes are standardized on the national mean, the negative values for the Heritage bilinguals indicate a performance somewhat below the national mean, while the Mixed bilinguals have scores just above the national mean. Likewise, the home literacy environment factors Book exposure and Literacy activities are standardized values, based on a sample including monolinguals. Heritage bilinguals had Book exposure values well below the mean of 0 for the overall sample.

**Table 2:** Means and standard deviations for outcome and predictor variables for each bilingual group; *p*-value and *η*^2^ effect size for group differences (ANOVA).

	Heritage bilinguals		Mixed bilinguals		Group difference	
	Mean	SD	Mean	SD	*p*	*η* ^2^
*2^nd^ grade Language and literacy outcomes*						
Language comprehension	−0.67	1.14	0.04	0.81	<0.001	0.11
Decoding	−0.23	1.04	0.16	0.50	<0.001	0.04
Reading comprehension	−0.35	1.02	0.11	0.85	<0.001	0.05
*Predictors*						
Book exposure	−1.35	1.20	−0.01	1.05	<0.001	0.26
Literacy activities	−0.20	0.91	−0.09	1.01	=0.143	0.00
Maternal Danish-language skills	3.9	1.1	4.6^1^	0.8	<0.001	0.12
Maternal Danish-language use	2.7	1.0	3.8^2^	1.1	<0.001	0.21
Paternal Danish-language skills	3.6	1.2	4.4^3^	1.1	<0.001	0.09
Paternal Danish-language use	2.6	1.2	3.9^4^	1.3	<0.001	0.21
Child’s Danish-language use at home	3.4	1.0	4.3	0.8	<0.001	0.21
Child’s Danish-language use in childcare	4.7	0.7	4.9	0.4	<0.001	0.05
Child’s Danish-language use with friends	3.8	1.1	4.5	0.8	<0.001	0.15

1 The means were 5.0 (0.3) for native Danish mothers and 4.3 (0.91) for nonnative mothers.

2 The means were 4.5 (0.7) for native Danish mothers and 3.4 (1.11) for nonnative mothers.

3 The means were 4.8 (0.7) for native Danish fathers and 3.7 (1.4) for nonnative fathers.

4 The means were 4.4 (1.1) for native Danish fathers and 3.1 (1.4) for nonnative fathers.

Note that, for the Mixed bilinguals, the mean values for maternal and paternal Danish-language skills and use are based on one native Danish parent and one nonnative parent. In four notes under Table 2, mean values are given for the native Danish and nonnative parent in those families. The mean value for the native Danish mothers’ and fathers’ Danish-language skills were unsurprisingly near the ceiling value (5), whereas their degrees of Danish-language use were a little lower. This indicates some degree of use of the partner’s heritage language. The nonnative parent in the Mixed bilingual group had generally higher Danish-language skills and use than the nonnative parents in the Heritage bilingual group, except for paternal Danish-language skills, which were about the same in the two groups.

The two groups of bilinguals differed significantly on all but one variable, with Mixed bilinguals having higher 2^nd^ grade Danish-language and reading scores, more supportive home literacy environments during the preschool years, higher own use of Danish in the preschool years, and higher parental use of Danish as well as higher parental Danish skills, according to self-report. Only for the extent of preschool literacy activities were the two bilingual groups similar.

As an initial examination of the relationship of our predictors to the outcomes, Table 3 shows zero-order correlations.

**Table 3:** Zero-order correlations of predictors to the three outcomes, language comprehension, decoding, and reading comprehension for the two groups of bilinguals combined.

	Lang. comp.	Decoding	Read. comp.
Maternal education	0.37	0.26	0.26
Household income	0.30	0.22	0.20
Book exposure	0.41	0.30	0.33
Literacy activities	0.02	0.01	0.03
Maternal Danish-language skills	0.26	0.18	0.18
Maternal Danish-language use	0.23	0.11	0.12
Paternal Danish-language skills	0.16	0.14	0.13
Paternal Danish-language use	0.16	0.13	0.09
Child’s Danish-language use at home	0.28	0.13	0.12
Child’s Danish-language use in childcare	0.20	0.13	0.10
Child’s Danish-language use with friends	0.31	0.17	0.14

All correlations were statistically significant (*p*s < 0.001), except for the correlations involving the literacy activities factor (*p*s > 0.400).

All predictors, except for the literacy activities factor, were significantly correlated with the three outcomes. Among the two SES predictors, maternal education coefficients were slightly higher than those for household income. Among the home literacy environment predictors, book exposure was clearly more strongly correlated with outcomes than literacy activities were. Among the language use and skills predictors, maternal Danish use and skills as well as the child’s own Danish use patterns were more strongly correlated with the outcomes than paternal skills and use were. Changing the perspective to the three outcomes in 2^nd^ grade, language comprehension was generally more strongly correlated with the predictors than decoding and reading comprehension were.

The correlations were generally weak to moderate, but of similar magnitude to comparable correlations previously found—for example, maternal education with child reading (*r* = 0.23, *p* < 0.05), or number of books in the home with child reading (*r* = 0.36, *p* < 0.001) in same-age native Dutch monolingual children (van Bergen et al., 2017). Therefore, each variable explains on a small part of the variance in the outcome variables, Book exposure being the most potent predictor, explaining 17% (0.41^2^) of the variance in language comprehension.

Recall that the children were originally sample for two intervention studies (see section 2.1). We correlated a binary variable for participation in the control or an intervention group in childcare with the three outcomes in 2^nd^ grade. The correlation was around 0 and nonsignificant in all three cases (language comprehension, *r* = −0.02, *p* = 0.560; decoding, *r* = −0.03, *p* = 0.404; reading comprehension, *r* = −0.01, *p* = 0.835). Although there may be small differences in the long-term effect of the different intervention arms, we consider those differences unlikely to influence the present results, and, for parsimony, we do not include the intervention variable in the below models (except for in a robustness check, see below).

### 3.2. Predicting bilinguals’ 2^nd^ grade Danish-language and reading outcomes

Our first question was how heritage language vs. majority language use patterns before school start predict bilingual students’ Danish majority language and reading skills in 2^nd^ grade. We wanted to determine the extent to which language use patterns explain variance in majority language and reading outcomes after accounting for variance related to bilingual group (Heritage vs. Mixed bilingualism), SES, and home literacy environment quality. Therefore, we estimated three series of hierarchical regression models, one for each of the three outcomes. For each series, blocks of predictors were entered in four stages. Stage 1: Bilingual group. Stage 2: SES. Stage 3: Home literacy environments. Stage 4: Child and parent use of heritage language vs. Danish use, and parent Danish language skills.

All 12 models were statistically significant (*p*s < 0.001). Tables 4–6 show, for each of the three outcomes, how much variance is explained at each stage, how much additional variance is accounted for by entering new predictors at each stage, as well as coefficients for individual predictors at each stage.

**Table 4:** Four-stage hierarchical regression model of 2^nd^ grade language comprehension scores predicted by type of bilingualism (Heritage is reference category), SES, home literacy environments before entering school, and child use and parental skill and use of Danish before entering school.

Language comprehension model		*β*	*SE*	*p*	*R* ^2^	*ΔR* ^2^
Stage 1	Type of bilingualism	0.81	0.11	0.001	0.14	
Stage 2	Type of bilingualism	0.53	0.10	0.001	0.21	0.07***
	Maternal education	0.19	0.04	0.001		
	Household income	0.19	0.06	0.002		
Stage 3	Type of bilingualism	0.33	0.10	0.001	0.25	0.04***
	Maternal education	0.10	0.05	0.038		
	Household income	0.13	0.06	0.027		
	Book exposure	0.23	0.04	0.001		
	Literacy activities	−0.00	0.05	1.000		
Stage 4	Type of bilingualism	0.21	0.10	0.041	0.29	0.04***
	Maternal education	0.09	0.05	0.075		
	Household income	0.10	0.06	0.083		
	Book exposure	0.19	0.04	0.001		
	Literacy activities	−0.01	0.04	0.853		
	Maternal Danish-language skills	0.08	0.07	0.219		
	Maternal Danish-language use	−0.01	0.06	0.873		
	Paternal Danish-language skills	0.14	0.07	0.032		
	Paternal Danish-language use	−0.13	0.07	0.059		
	Child’s Danish-language use at home	0.07	0.08	0.473		
	Child’s Danish-language use in childcare	0.06	0.08	0.578		
	Child’s Danish-language use with friends	0.16	0.08	0.035		

*** *p* < 0.001.

**Table 5:** Four-stage hierarchical regression model of 2^nd^ grade decoding scores predicted by type of bilingualism (Heritage is reference category), SES, home literacy environments before entering school, and child use and parental skill and use of Danish before entering school.

Decoding model		*β*	*SE*	*p*	*R* ^2^	*ΔR* ^2^
Stage 1	Type of bilingualism	0.42	0.09	0.001	0.047	
Stage 2	Type of bilingualism	0.20	0.10	0.039	0.10	0.05***
	Maternal education	0.15	0.04	0.001		
	Household income	0.15	0.06	0.012		
Stage 3	Type of bilingualism	0.05	0.10	0.658	0.13	0.03***
	Maternal education	0.08	0.04	0.071		
	Household income	0.10	0.06	0.083		
	Book exposure	0.18	0.05	0.001		
	Literacy activities	0.00	0.04	0.957		
Stage 4	Type of bilingualism	0.01	0.11	0.962	0.15	0.02
	Maternal education	0.08	0.04	0.079		
	Household income	0.10	0.06	0.130		
	Book exposure	0.16	0.05	0.001		
	Literacy activities	−0.00	0.04	0.998		
	Maternal Danish-language skills	0.02	0.06	0.697		
	Maternal Danish-language use	−0.05	0.06	0.475		
	Paternal Danish-language skills	0.10	0.06	0.073		
	Paternal Danish-language use	−0.01	0.07	0.824		
	Child’s Danish-language use at home	−0.03	0.08	0.706		
	Child’s Danish-language use in childcare	0.08	0.08	0.455		
	Child’s Danish-language use with friends	0.08	0.08	0.294		

*** *p* < 0.001.

**Table 6:** Four-stage hierarchical regression model of 2^nd^ grade reading comprehension scores predicted by type of bilingualism (Heritage is reference category), SES, home literacy environments before entering school, and child use and parental skill and use of Danish before entering school.

Reading comprehension model		*β*	*SE*	*p*	*R* ^2^	*ΔR* ^2^
Stage 1	Type of bilingualism	0.49	0.09	0.001	0.06	
Stage 2	Type of bilingualism	0.31	0.10	0.003	0.10	0.04***
	Maternal education	0.14	0.04	0.001		
	Household income	0.10	0.06	0.082		
Stage 3	Type of bilingualism	0.12	0.10	0.229	0.15	0.05***
	Maternal education	0.06	0.04	0.181		
	Household income	0.04	0.06	0.427		
	Book exposure	0.22	0.04	0.001		
	Literacy activities	0.02	0.03	0.671		
Stage 4	Type of bilingualism	0.12	0.10	0.216	0.16	0.01
	Maternal education	0.06	0.04	0.195		
	Household income	0.05	0.06	0.431		
	Book exposure	0.20	0.05	0.001		
	Literacy activities	0.01	0.04	0.748		
	Maternal Danish-language skills	−0.00	0.06	0.947		
	Maternal Danish-language use	0.01	0.07	0.856		
	Paternal Danish-language skills	0.13	0.06	0.031		
	Paternal Danish-language use	−0.13	0.07	0.062		
	Child’s Danish-language use at home	0.00	0.08	0.977		
	Child’s Danish-language use in childcare	0.02	0.08	0.788		
	Child’s Danish-language use with friends	0.04	0.07	0.605		

*** *p* < 0.001.

Table 4 shows the 2^nd^ grade language comprehension estimates. As expected, the stage 1 model, with just bilingual group as a predictor, reveals that Mixed bilinguals have higher scores than Heritage bilinguals do. Adding SES predictors (stage 2) significantly increased variance explained by 7%, home literacy predictors (stage 3) explained an additional significant 4%, and language use patterns (stage 4) yet an additional significant 4%.

Table 5 shows the 2^nd^ grade decoding estimates. Aga in, the stage 1 model, reveals a substantial bilingual group difference in favor of Mixed bilingualism, but the group coefficient for decoding was only half the size of the group coefficient found for language comprehension. Adding SES predictors significantly increased variance explained by 5%, home literacy predictors explained an additional significant 3%, but while language use patterns explained an additional 2%, this addition was not statistically significant.

Table 6 shows the 2^nd^ grade reading comprehension estimates. Again, the stage 1 model, reveals a substantial bilingual group difference in favor of Mixed bilingualism, but the group coefficient for reading comprehension was much smaller than for language comprehension. Adding SES predictors increased variance explained by 4%, home literacy predictors explained an additional 5%, but while language use patterns explained an additional 1%, this addition was not statistically significant.

In summary, the full model of 2^nd^ grade Danish language comprehension explained 29% of the variance. For decoding and reading comprehension, the full models explained less variance, namely 15 and 16%. The pattern of results that emerged from the four-stage models is that heritage language use frequency and parental Danish-language skills in the preschool years explained a small but significant part of the variance in bilingual children’s 2^nd^ grade Danish language comprehension skills after having accounted for type of bilingualism, SES, and home literacy environments; however, this was not the case for the decoding and reading comprehension outcomes. As a robustness check, we estimated models similar to those in Tables 4–6 with the above-mentioned binary predictor indicating whether the children had been in a control group or an intervention group (that is, not differentiating between the type of intervention group). The variable indicating intervention was entered at stage 1. The added intervention group variable explained no variance in the 2nd grade outcomes on its own (*R*^2^ = 0.000–0.001), and accordingly did not change the results of the models reported.

Our second research question asked whether relations of parental majority vs. heritage language use to child language and reading outcomes differ significantly between Heritage and Mixed bilinguals. Recall that, not surprisingly, mean levels of Danish use in the home were significantly higher (and levels of heritage language use lower) among parents of Mixed bilinguals than among parents of Heritage bilinguals. Additionally, Mixed bilinguals had significantly higher Danish language and reading scores in 2^nd^ grade, which could be causally related to more Danish exposure in the home before entering school. But at the same time, Mixed bilinguals also had parents with higher SES and had better home literacy environments than Heritage bilinguals. To determine if type of bilingualism moderated effects of parental use of Danish vs. the heritage language when controlling for SES and home literacy environment quality, we estimated follow-up models which had interaction terms for both maternal and paternal Danish use × bilingual group. Apart from the interaction terms, the models were identical to the above stage 4 model for each of the three outcomes. However, neither of the follow-up models revealed significant interactions (*p*-values between 0.063 and 0.934). The one interaction approaching significance (*p* = 0.063) was a trend toward a positive relationship of more maternal Danish use to 2^nd^ grade language comprehension in Heritage bilinguals, which was not found in Mixed bilinguals. However, given that we examined six interaction terms (three outcomes, both maternal and paternal language use in interaction with bilingual group) in order to answer essentially the same question, Bonferroni corrections are probably appropriate, in which case no interaction approached significance. Accordingly, we conclude that the relations of parental heritage language vs. majority language use did not differ significantly between Mixed and Heritage bilinguals. The full interaction models are provided in Supplementary material.

Research question 3 asked whether the relationship of parental Danish-language use to children’s outcomes is modified by parental Danish skills. We addressed this question by estimating follow-up models which had interaction terms for both maternal and paternal Danish use × Danish skills but were otherwise identical to the above stage 4 model for each of the three outcomes. However, neither of the follow-up models revealed significant interactions (*p*-values between 0.148 and 0.902); that is, parental Danish language skill did not significantly moderate the relationship of degree of Danish use to child language and reading outcomes.

Having addressed our three specific research questions, we now explore how individual predictors relate to bilingual children’s 2^nd^ grade outcomes. The large effect of type of bilingualism—indicating an advantage of Mixed over early Heritage bilingualism—is substantially reduced for all three outcomes when adding SES, home literacy environment and language use patterns as controls, and remains significant only for language comprehension. In other words, for decoding and reading comprehension in 2^nd^ grade, Mixed bilingualism in itself does not give a significant advantage over Heritage bilingualism, but does so for language comprehension. The models suggest that the substantial, real world mean difference in 2^nd^ grade outcomes between the two groups of bilinguals (see Table 2) is largely explained by SES, home literacy environments and, for language comprehension, language use patterns, rather than whether or not the children had access Danish-language exposure from a native parent in their home from the beginning of life. Note in this regard, however, that only the home literacy environment factor, book exposure, was systematically related to the three outcomes with statistical significance, pointing to this factor as a central predictor of later majority language and reading skills in bilingual children.

Turning to the SES variables, it is remarkable that neither maternal education nor household income was significantly associated with the outcomes when controlling for home literacy environments and children’s language use and parental language use and skills (stage 4 model). In fact, SES relations to the two reading outcomes, decoding and reading comprehension, were nonsignificant already in the stage 3 models with the addition of home literacy environment factors. This suggests that especially book exposure (number of books, library visits, frequency of reading and reading from a young age) is an important factor underlying the often-seen differential outcomes in children of high vs. low SES parents.

## 4. Discussion

Our main question was whether degree of relative use of the heritage language and majority language in the home of preschool-age bilingual children and parental majority skills explain a significant amount of variance in their 2^nd^ grade majority language and reading skills after controlling for type of bilingualism (Heritage vs. Mixed), family SES and home literacy environment quality. We found that the answer differs depending on the outcome. Relative use of the heritage language and the majority language, Danish, explained variance in 2^nd^ grade Danish language comprehension scores; specifically, more use of Danish in the preschool years was a positive predictor of 2^nd^ grade Danish language comprehension. On the other hand, relative language use did not explain variance in the two reading outcomes, decoding and reading comprehension in Danish. The relations between parent’s and children’s own language use and later outcomes were not significantly moderated by type of bilingualism (Mixed vs. Heritage) or by self-reported Danish-language parental skill. Controlling for covariates in a statistical model naturally does not undo real world differences such as those between Mixed vs. Heritage language bilingual children (corresponding approximately to simultaneous vs. sequential bilingual children in other research). However, we find it interesting and important that degree of majority language use is similarly related—or unrelated—to child language and reading outcomes in both groups of bilinguals.

Recall that the language comprehension measure was presented in written format and therefore also required basic reading skills. Therefore, one might argue that it is really a reading comprehension measure. However, the finding that home language use and skill measures explained variance in the language comprehension outcomes but not the two reading outcomes, indicates to us that the two tests do measure different skills.

The results are consistent previous research discussed in the introduction which found that parent’s and children’s relative use of the heritage and majority language is related to later language but not reading outcomes. Our finding that the relative language use in the preschool years did not explain variance in 2^nd^ grade decoding and reading comprehension skills is extends the finding Winsler et al. (2014), who found that language use was not significantly related to early literacy skills in kindergarten. However, relative use of the heritage language and the Danish majority language in the preschool years did explain variance in 2^nd^ grade Danish language comprehension skills. This result is consistent with those of Thordardottir (2019) namely that relative use of heritage and majority language was related to vocabulary size, that is, another type of oral language skill that than that examined in the present study.

Overall, the finding of a positive effect of parent’s and children’s own relative use of the majority and heritage language for 2^nd^ grade language comprehension is consistent with a line of research that converges on the view that bilingual language proficiency is different than monolingual language proficiency in that each language develops in response to usage of each language (Ellis, 2002; Hernandez et al., 2005; MacWhinney, 2005), and that bilinguals should not be expected to perform as monolinguals in each of their languages (Grosjean, 1989). However, even though bilingual children may draw on bilingual resources, oral-language proficiency in the majority language used in school is necessary for successful educational outcomes (e.g., Demie and Strand, 2006; Halle et al., 2012). Our findings are also consistent with the view that reading skills should be little or at least less influenced by relative use of each language in bilinguals, because reading-related skills transfer better between languages than do oral language skills (Cummins, 1991; Adesope et al., 2010; Hammer et al., 2014).

Research question 2 and 3 asked whether relations of parental language use to child outcomes were moderated by type of bilingualism (Mixed vs. Heritage) or by parental majority language skill. The questions are in some sense related in that majority language skill is higher in the parents in Mixed bilingual families. Moderation analyses for both questions revealed nonsignificant interactions. This is a somewhat surprising result because it would be reasonable to suppose that majority language input is more helpful when the parent providing the input is a more proficient speaker. However, on this note, there were trends and sometimes just significant coefficients pointing to a *negative* influence of more paternal use of the majority language and a *positive* influence of higher paternal majority language skill (Tables 4–6). The positive influence makes immediate sense. However, we speculate that a negative influence of paternal majority could arise when fathers withhold richer heritage language input in order to—with the best of intentions—support majority language development in the child by speaking the majority language to the best of their abilities, even when not proficient. However, these results and their interpretation should be regarded with caution because the *p*-values for the relations are just above or just below 0.05. Additionally, the use and skill variables are based on parent’s own report, which could be biased.

A minor, but potentially important finding is that the child’s own majority language use with friends (outside of the home and outside childcare) was a significant positive predictor of language comprehension (Table 4). We speculate that when immigrant children use the majority language with friends, this would often be native speakers of the majority language, who can be an additional rich source of majority-language input. Alternatively, or additionally, a high degree of majority language use with friends may be an indicator of a generally high degree of assimilation in the host country society, which could be associated with a favorable majority language development.

Finally, among the predictors of bilingual 2^nd^ graders’ language and reading outcomes in the present study, it is noteworthy that the home literacy factor book exposure was the only persistently significant predictor in models with multiple other predictors. Moreover, SES predictors of decoding and reading comprehension became nonsignificant when accounting for differences in home literacy environments (stage 3 of the models in Tables 5, 6). This suggests that an early start and a high frequency of book reading in early childhood is a highly supportive activity for language development and reading in school, irrespective of other factors such as type of bilingualism (Heritage or Mixed), SES and relative use of the heritage and majority language, and that differences in home literacy environments importantly account for the often-seen SES relationship to language development, which supports previous research indicating that typical SES measures are surface to underlying variables associated with SES (e.g., Singh et al., 2022).

### 4.1. Limitations and implications

The study had certain limitations worth noting. Scores for language use patterns in the home as well as home literacy environments are based on parent report rather than direct observation. This means that the scores could be influenced by social desirability, and their statistical relations to the outcomes could be underestimated. Importantly, we did not obtain measures or estimates of *absolute* language use with the children. That is, for example, a mother who speaks an equal amount of heritage and majority language to the child would have a score of 3 for language use (indicating 50/50 use) no matter whether she has very few or very many interactions with her child every day. In addition, although native speakers may vary in native language skill—especially bilinguals—the self-rating of Danish-language skills may be a conceptually different task for native and nonnative speakers. We did not obtain a measure of children’s heritage language skills, which would have strengthened our argument that an important early base of later second-language and reading development, is a favorable early language learning environment *in general* rather than an early focus on second-language input. Likewise, we did not obtain measures of literacy skills in the heritage language. In addition, our measure of parent education is less reliable than measures of income, because the national register in Denmark only has reliable measures of education taken in Denmark (but recall that host country education has been found to be indicative of majority language skills, Hoff et al., 2018). It is also a limitation that our sample was biased towards higher SES than the general population of bilingual children in Denmark, as noted in section 2.1. Finally, since this is a longitudinal study, our first measures, those of the home literacy environment, were sampled quite a while ago, namely in 2013. Since then, the digital aspect of the home literacy environment has surely become more prominent, which means that our results with regard to home literacy environments may not generalize to present-day home literacy environments. Therefore, there is clearly a need for more research taking digital aspects of the home literacy environment into account. This new line of research may prove especially interesting and important with regard to bilingual children. This is because they often grow up in a context where children’s books and printed literacy materials in the heritage language are not easily available, if available at all. However, the digital modality may offer a means to reduce this problem.

We would like to conclude by pointing out three important implications of our results. (1) 2^nd^ grade majority language outcomes in the heritage bilingual children were substantially below the national means, and the degree of use and quality of the majority language in the home before school start explained part of the variance. This is not to say that parents who do not speak the majority language well should nevertheless speak it with their child; these parents can provide richer input in the heritage language (Hoff et al., 2020). However, it is important to ensure that bilingual children with little majority language exposure in the home are offered the opportunity to realize their potential for majority language acquisition, for example, in childcare or rather more informal majority language contexts such as playing with friends who are native speakers of the majority language.

(2) Reading development during bilingual children’s early years of school does not seem to be significantly impacted by parent’s and children’s own relative use of the heritage language and majority language in the home during the preschool years. This suggests that an important foundation for bilingual children’s reading skills later in school is a stimulating home literacy environment which starts them on a favorable language development trajectory from the very early years of life—independently of whether language use in the home leans more towards heritage language or majority language use. The implication of this is that language professionals should make clear to parents that they should interact with their bilingual children and stimulate their language development in whatever language it feels most natural to do so.

(3) A stimulating home literacy environment, notably an early start and a high frequency of shared book reading, is an important protective factor for reading development in bilingual children in majority-language schools.

## Data availability statement

The data analyzed in this study is subject to the following licenses/restrictions: The data resides in a repository in Statistics Denmark and cannot be exported. Interested researchers may be granted access to the data on the Statistics Denmark server upon request to Mette Vad Andersen. Requests to access these datasets should be directed to mvandersen@econ.au.dk.

## Ethics statement

Ethical review and approval was not required for the study on human participants in accordance with the local legislation and institutional requirements. Written informed consent to participate in this study was provided by the participants’ legal guardian/next of kin.

## Author contributions

AH conceived the idea for this research, took part in data collection for the original RCTs contributing the data, carried out analyses, and wrote a draft of the article. DB was PI in the original RCTs and took part in data collection. For the present study she contributed analysis ideas, ideas for organizing the article, and commented and revised the draft. All authors contributed to the article and approved the submitted version.

## Funding

This research was supported by a grant to AH by the TrygFonden via TrygFonden’s Center for Child Research (AU ID no. 31174).

## Conflict of interest

The authors declare that the research was conducted in the absence of any commercial or financial relationships that could be construed as a potential conflict of interest.

## Publisher’s note

All claims expressed in this article are solely those of the authors and do not necessarily represent those of their affiliated organizations, or those of the publisher, the editors and the reviewers. Any product that may be evaluated in this article, or claim that may be made by its manufacturer, is not guaranteed or endorsed by the publisher.
