# Supplementary material for: Relative heritage language and majority language use before school start explains variance in 2nd grade majority language but not reading skills
Source: Front Psychol. 2023 Apr 17;14:1134830. doi: 10.3389/fpsyg.2023.1134830 (PMC10150131; doi:10.3389/fpsyg.2023.1134830)
Supplement: Supplementary file 1 [file Data_Sheet_1.docx]

Supplementary material

**Interaction models addressing research question 2 for each of three outcomes**

Table S-1. Regression model predicting 2^nd^ grade language comprehension scores with interaction terms for Type of bilingualism × Maternal Danish-language use and Type of bilingualism × Paternal Danish-language use (otherwise similar to the stage 4 model in Table 4). Mixed bilinguals is reference category.

| Language comprehension model | *β* | *SE* | *p* |
| --- | --- | --- | --- |
| Type of bilingualism | –0.22 | 0.10 | 0.039 |
| Maternal education | 0.09 | 0.05 | 0.104 |
| Household income | 0.10 | 0.06 | 0.072 |
| Book exposure | 0.19 | 0.04 | 0.001 |
| Literacy activities | –0.00 | 0.04 | 0.864 |
| Maternal Danish-language skills | 0.08 | 0.07 | 0.253 |
| Paternal Danish-language skills | 0.14 | 0.06 | 0.018 |
| Maternal Danish-language use | –0.07 | 0.07 | 0.317 |
| Type Bilingualism × Mat. Danish-lang. use | 0.22 | 0.12 | 0.063 |
| Paternal Danish-language use | –0.14 | 0.07 | 0.036 |
| Type Bilingualism × Pat. Danish-lang. use | –0.04 | 0.12 | 0.771 |
| Child’s Danish-language use at home | 0.03 | 0.08 | 0.677 |
| Child’s Danish-language use in childcare | 0.04 | 0.08 | 0.643 |
| Child’s Danish-language use with friends | 0.17 | 0.08 | 0.030 |

Table S-2. Regression model predicting 2^nd^ grade decoding scores with interaction terms for Type of bilingualism × Maternal Danish-language use and Type of bilingualism × Paternal Danish-language use (otherwise similar to the stage 4 model in Table 5). Mixed bilinguals is reference category.

| Decoding model | *β* | *SE* | *p* |
| --- | --- | --- | --- |
| Type of bilingualism | 0.00 | 0.11 | 0.964 |
| Maternal education | 0.08 | 0.05 | 0.097 |
| Household income | 0.10 | 0.06 | 0.123 |
| Book exposure | 0.16 | 0.05 | 0.001 |
| Literacy activities | 0.00 | 0.04 | 0.997 |
| Maternal Danish-language skills | 0.02 | 0.06 | 0.723 |
| Paternal Danish-language skills | 0.10 | 0.06 | 0.061 |
| Maternal Danish-language use | –0.07 | 0.07 | 0.319 |
| Type Bilingualism × Mat. Danish-lang. use | 0.08 | 0.11 | 0.466 |
| Paternal Danish-language use | –0.02 | 0.07 | 0.808 |
| Type Bilingualism × Pat. Danish-lang. use | –0.01 | 0.10 | 0.886 |
| Child’s Danish-language use at home | –0.04 | 0.08 | 0.643 |
| Child’s Danish-language use in childcare | 0.05 | 0.08 | 0.478 |
| Child’s Danish-language use with friends | 0.08 | 0.08 | 0.274 |

Table S-3. Regression model predicting 2^nd^ grade reading comprehension scores with interaction terms for Type of bilingualism × Maternal Danish-language use and Type of bilingualism × Paternal Danish-language use (otherwise similar to the stage 4 model in Table 6). Mixed bilinguals is reference category.

| Reading comprehension model | *β* | *SE* | *p* |
| --- | --- | --- | --- |
| Type of bilingualism | –0.13 | 0.10 | 0.218 |
| Maternal education | 0.05 | 0.05 | 0.232 |
| Household income | 0.05 | 0.06 | 0.415 |
| Book exposure | 0.20 | 0.05 | 0.001 |
| Literacy activities | 0.01 | 0.04 | 0.743 |
| Maternal Danish-language skills | –0.01 | 0.07 | 0.915 |
| Paternal Danish-language skills | 0.14 | 0.06 | 0.024 |
| Maternal Danish-language use | –0.01 | 0.07 | 0.839 |
| Type Bilingualism × Mat. Danish-lang. use | 0.09 | 0.10 | 0.362 |
| Paternal Danish-language use | –0.14 | 0.07 | 0.060 |
| Type Bilingualism × Pat. Danish-lang. use | –0.01 | 0.10 | 0.934 |
| Child’s Danish-language use at home | –0.01 | 0.08 | 0.933 |
| Child’s Danish-language use in childcare | 0.02 | 0.08 | 0.820 |
| Child’s Danish-language use with friends | 0.05 | 0.07 | 0.571 |

**Interaction models addressing research question 3 for each of three outcomes**

Table S-4. Regression model predicting 2^nd^ grade language comprehension scores with interaction terms for Maternal Danish-language skills × Maternal Danish-language use and Paternal Danish-language skills × Paternal Danish-language use (otherwise similar to the stage 4 model in Table 4).

| Language comprehension model | *β* | *SE* | *p* |
| --- | --- | --- | --- |
| Type of bilingualism | –0.28 | 0.11 | 0.010 |
| Maternal education | 0.09 | 0.05 | 0.073 |
| Household income | 0.10 | 0.06 | 0.080 |
| Book exposure | 0.19 | 0.04 | 0.001 |
| Literacy activities | –0.00 | 0.04 | 0.914 |
| Maternal Danish-language skills | 0.05 | 0.07 | 0.463 |
| Maternal Danish-language use | –0.01 | 0.07 | 0.855 |
| Mat. Danish-lang. skills × Danish-lang. use | –0.04 | 0.06 | 0.478 |
| Paternal Danish-language skills | 0.09 | 0.07 | 0.171 |
| Paternal Danish-language use | –0.12 | 0.07 | 0.074 |
| Pat. Danish-lang. skills × Danish-lang. use | –0.09 | 0.06 | 0.148 |
| Child’s Danish-language use at home | 0.07 | 0.08 | 0.324 |
| Child’s Danish-language use in childcare | 0.03 | 0.08 | 0.665 |
| Child’s Danish-language use with friends | 0.15 | 0.08 | 0.042 |

Table S-5. Regression model predicting 2^nd^ grade decoding scores with interaction terms for Maternal Danish-language skills × Maternal Danish-language use and Paternal Danish-language skills × Paternal Danish-language use (otherwise similar to the stage 4 model in Table 5).

| Decoding model | *β* | *SE* | *p* |
| --- | --- | --- | --- |
| Type of bilingualism | –0.03 | 0.11 | 0.791 |
| Maternal education | 0.08 | 0.04 | 0.090 |
| Household income | 0.10 | 0.06 | 0.121 |
| Book exposure | 0.16 | 0.05 | 0.001 |
| Literacy activities | 0.00 | 0.04 | 0.984 |
| Maternal Danish-language skills | –0.02 | 0.07 | 0.808 |
| Maternal Danish-language use | –0.03 | 0.07 | 0.625 |
| Mat. Danish-lang. skills × Danish-lang. use | –0.06 | 0.06 | 0.259 |
| Paternal Danish-language skills | 0.09 | 0.07 | 0.154 |
| Paternal Danish-language use | –0.02 | 0.07 | 0.805 |
| Pat. Danish-lang. skills × Danish-lang. use | –0.09 | 0.06 | 0.148 |
| Child’s Danish-language use at home | –0.03 | 0.08 | 0.713 |
| Child’s Danish-language use in childcare | 0.05 | 0.08 | 0.468 |
| Child’s Danish-language use with friends | 0.07 | 0.08 | 0.336 |

Table S-6. Regression model predicting 2^nd^ grade reading comprehension scores with interaction terms for Maternal Danish-language skills × Maternal Danish-language use and Paternal Danish-language skills × Paternal Danish-language use (otherwise similar to the stage 4 model in Table 6).

| Reading comprehension model | *β* | *SE* | *p* |
| --- | --- | --- | --- |
| Type of bilingualism | –0.17 | 0.10 | 0.110 |
| Maternal education | 0.06 | 0.04 | 0.203 |
| Household income | 0.05 | 0.06 | 0.419 |
| Book exposure | 0.20 | 0.05 | 0.001 |
| Literacy activities | 0.01 | 0.04 | 0.712 |
| Maternal Danish-language skills | –0.04 | 0.07 | 0.621 |
| Maternal Danish-language use | 0.02 | 0.07 | 0.804 |
| Mat. Danish-lang. skills × Danish-lang. use | –0.05 | 0.06 | 0.392 |
| Paternal Danish-language skills | 0.11 | 0.07 | 0.113 |
| Paternal Danish-language use | –0.13 | 0.07 | 0.070 |
| Pat. Danish-lang. skills × Danish-lang. use | –0.04 | 0.06 | 0.507 |
| Child’s Danish-language use at home | 0.01 | 0.08 | 0.886 |
| Child’s Danish-language use in childcare | 0.02 | 0.08 | 0.841 |
| Child’s Danish-language use with friends | 0.03 | 0.07 | 0.665 |
